# Supplementary material for: Discussing spiritual health in primary care and the HOPE tool—A mixed methods survey of GP views
Source: PLoS One. 2022 Nov 8;17(11):e0276281. doi: 10.1371/journal.pone.0276281 (PMC9642893; doi:10.1371/journal.pone.0276281)
Supplement: Attachment — Submitted filename: plos one.docx [file pone.0276281.s002.docx]

Abstract (results): please correct the percentage of GPs responders in 49.71

Introduction: line 48. When you talking about the importance of spirituality in patients please check studies in neurology and in oncology that have proved the importance of spirituality in some disease ( Sparaco M, Miele G, Abbadessa G, Ippolito D, Trojsi F, Lavorgna L, Bonavita S. Correction to: Pain, quality of life, and religiosity in people with multiple sclerosis. Neurol Sci. 2021 Dec 10. doi: 10.1007/s10072-021-05814-x. Epub ahead of print. Erratum for: Neurol Sci. 2021 Nov 23;: PMID: 34890003.)

Discussion: in which other studies the structured tool (HOPE) was used ?
