# Supplementary material for: Discussing spiritual health in primary care and the HOPE tool—A mixed methods survey of GP views
Source: PLoS One. 2022 Nov 8;17(11):e0276281. doi: 10.1371/journal.pone.0276281 (PMC9642893; doi:10.1371/journal.pone.0276281)
Supplement: Attachment — Submitted filename: response to reviewers HOPE paper 28th June mk1.docx [file pone.0276281.s003.docx]

Thank you for very much for your review of our paper: Discussing spiritual health in primary care and the HOPE tool- A mixed methods survey of GP views

Journal Requirements:

When submitting your revision, we need you to address these additional requirements.

1. Please ensure that your manuscript meets PLOS ONE's style requirements, including those for file naming. The PLOS ONE style templates can be found at 
<https://journals.plos.org/plosone/s/file?id=wjVg/PLOSOne_formatting_sample_main_body.pdf> and 
<https://journals.plos.org/plosone/s/file?id=ba62/PLOSOne_formatting_sample_title_authors_affiliations.pdf>.

Thank you, those links are very clear. The style has been revised throughout, including the authors’ affiliation.

2. Please provide additional details regarding participant consent. In the ethics statement in the Methods and online submission information, please ensure that you have specified what type you obtained (for instance, written or verbal, and if verbal, how it was documented and witnessed). If your study included minors, state whether you obtained consent from parents or guardians. If the need for consent was waived by the ethics committee, please include this information.

Thank you, to clarify this, we have added “in writing”, and the statement now reads: “Consent was taken online **in writing,** prior to the start of the online survey.” There were no children involved in the study, only qualified General Practitioners in England.

3. We noted in your submission details that a portion of your manuscript may have been presented or published elsewhere. [The paper: "What do doctors understand by spiritual health? A survey of UK general practitioners" is related to this submission, in that the data were gathered in the same survey, and therefore table 1 in both papers is the same. That paper analysed data on how the participants defined "spiritual health", whereas this paper analyses the data about use of the hope tool, and discussing spiritual health in the consultation. While there is minimal overlap, data from different questions within the survey are presented in the two different papers, and therefore this is not dual publication of the same data. ]

Please clarify whether this publication was peer-reviewed and formally published. If this work was previously peer-reviewed and published, in the cover letter please provide the reason that this work does not constitute dual publication and should be included in the current manuscript.

“What do doctors understand by spiritual health? A survey of UK general practitioners“ was peer reviewed, and formally published. (Whitehead O, Jagger C, Hanratty B. What do doctors understand by spiritual health? A survey of UK general practitioners. BMJ Open 2021;11(8):e045110. doi: 10.1136/bmjopen-2020-045110) The data was gathered in the same survey, and hence the demographic details in table 1 are the same. However, there is minimal overlap with “Discussing spiritual health in primary care and the HOPE tool- A mixed methods survey of GP views.”, as this paper addresses the data on the HOPE tool, rather than definitions of the term spiritual health, and therefore this does not constitute dual publication of the same data. This statement has been added to the cover letter.

4. In your Data Availability statement, you have not specified where the minimal data set underlying the results described in your manuscript can be found. PLOS defines a study's minimal data set as the underlying data used to reach the conclusions drawn in the manuscript and any additional data required to replicate the reported study findings in their entirety. All PLOS journals require that the minimal data set be made fully available. For more information about our data policy, please see <http://journals.plos.org/plosone/s/data-availability>.

Upon re-submitting your revised manuscript, please upload your study’s minimal underlying data set as either Supporting Information files or to a stable, public repository and include the relevant URLs, DOIs, or accession numbers within your revised cover letter. For a list of acceptable repositories, please see <http://journals.plos.org/plosone/s/data-availability#loc-recommended-repositories>. Any potentially identifying patient information must be fully anonymized.

Important: If there are ethical or legal restrictions to sharing your data publicly, please explain these restrictions in detail. Please see our guidelines for more information on what we consider unacceptable restrictions to publicly sharing data: <http://journals.plos.org/plosone/s/data-availability#loc-unacceptable-data-access-restrictions>. Note that it is not acceptable for the authors to be the sole named individuals responsible for ensuring data access.

We will update your Data Availability statement to reflect the information you provide in your cover letter.

This is human research participant data. Participants were asked to consent to anonymised data “becoming part of a data set which can be accessed by other users running other research studies at Newcastle University and in other organisations. These organisations may be universities, or NHS organisations. [This] information will only be used by organisations and researchers to conduct research.” The authors are concerned that this doesn’t include consent for public data sharing, only for further research in universities or NHS organisations. Data will be shared upon reasonable request to the authors. The sentence “Data Access: While participants were not consented to allow public sharing of this data, data is available upon reasonable request to the authors.“ has been added.

5. Please include your full ethics statement in the ‘Methods’ section of your manuscript file. In your statement, please include the full name of the IRB or ethics committee who approved or waived your study, as well as whether or not you obtained informed written or verbal consent. If consent was waived for your study, please include this information in your statement as well.

“Ethics approval was sought and obtained from Newcastle University on 27 February 2019.” Has been added to the methods.

6. Please ensure that you refer to Figure 1 in your text as, if accepted, production will need this reference to link the reader to the figure.

Figure 1 can be removed from the paper, as it adds little to the text at this stage, thank you.

7. Please review your reference list to ensure that it is complete and correct. If you have cited papers that have been retracted, please include the rationale for doing so in the manuscript text, or remove these references and replace them with relevant current references. Any changes to the reference list should be mentioned in the rebuttal letter that accompanies your revised manuscript. If you need to cite a retracted article, indicate the article’s retracted status in the References list and also include a citation and full reference for the retraction notice.

We have reviewed the reference list, and believe it to be complete and correct. No papers have to our knowledge been retracted.


Reviewers' comments:

Reviewer's Responses to Questions

**Comments to the Author**

1. Is the manuscript technically sound, and do the data support the conclusions?

The manuscript must describe a technically sound piece of scientific research with data that supports the conclusions. Experiments must have been conducted rigorously, with appropriate controls, replication, and sample sizes. The conclusions must be drawn appropriately based on the data presented.

Reviewer #1: Partly

2. Has the statistical analysis been performed appropriately and rigorously?

Reviewer #1: Yes

3. Have the authors made all data underlying the findings in their manuscript fully available?

The [PLOS Data policy](https://eur03.safelinks.protection.outlook.com/?url=http%3A%2F%2Fwww.plosone.org%2Fstatic%2Fpolicies.action%23sharing&data=05%7C01%7Corla.whitehead%40newcastle.ac.uk%7Cd42241b5cfb64995012308da5933db4f%7C9c5012c9b61644c2a91766814fbe3e87%7C1%7C0%7C637920377623501072%7CUnknown%7CTWFpbGZsb3d8eyJWIjoiMC4wLjAwMDAiLCJQIjoiV2luMzIiLCJBTiI6Ik1haWwiLCJXVCI6Mn0%3D%7C3000%7C%7C%7C&sdata=sxXyjAt8XNTLtHIs%2FtNIgjPs2FNvVxOuB2J4RIlgJBk%3D&reserved=0) requires authors to make all data underlying the findings described in their manuscript fully available without restriction, with rare exception (please refer to the Data Availability Statement in the manuscript PDF file). The data should be provided as part of the manuscript or its supporting information, or deposited to a public repository. For example, in addition to summary statistics, the data points behind means, medians and variance measures should be available. If there are restrictions on publicly sharing data—e.g. participant privacy or use of data from a third party—those must be specified.

Reviewer #1: Yes

4. Is the manuscript presented in an intelligible fashion and written in standard English?

PLOS ONE does not copyedit accepted manuscripts, so the language in submitted articles must be clear, correct, and unambiguous. Any typographical or grammatical errors should be corrected at revision, so please note any specific errors here.

Reviewer #1: Yes

5. Review Comments to the Author

Please use the space provided to explain your answers to the questions above. You may also include additional comments for the author, including concerns about dual publication, research ethics, or publication ethics. (Please upload your review as an attachment if it exceeds 20,000 characters)

Reviewer #1: Abstract (results): please correct the percentage of GPs responders in 49.71

This has been made more exact.

Introduction: line 48. When you talking about the importance of spirituality in patients please check studies in neurology and in oncology that have proved the importance of spirituality in some disease ( Sparaco M, Miele G, Abbadessa G, Ippolito D, Trojsi F, Lavorgna L, Bonavita S. Correction to: Pain, quality of life, and religiosity in people with multiple sclerosis. Neurol Sci. 2021 Dec 10. doi: 10.1007/s10072-021-05814-x. Epub ahead of print. Erratum for: Neurol Sci. 2021 Nov 23;: PMID: 34890003.)

Thank you for your interest in this topic. Spiritual health does appear to be an important aspect of patients’ health, in multifactorial ways. Much of the research in this area can be difficult to compare and analyse, as religiosity and spiritual health and similar concepts are often conflated. We have not sought to justify why spiritual health is an important aspect of health in this study- the Royal College and the regulator have stated that GPs in the UK should be able to address a patient’s spiritual health. We have also not sought to present a definition of spiritual health to participants, as participants were asked to define the term for themselves. (Presented in our other publication: Whitehead O, Jagger C, Hanratty B. What do doctors understand by spiritual health? A survey of UK general practitioners. BMJ Open 2021). This has allowed this survey to consider discussions about spiritual health in the broadest sense, including religiosity, spirituality, but encompassing whatever the term meant to the participant.

Discussion: in which other studies the structured tool (HOPE) was used ?

As far as we are aware, this is the first time the acceptability of the HOPE tool to practitioners has been formally assessed in this way. Editorials and teaching sessions have included the tool, often alongside FICA, and/or BELIEF. The benefits of the HOPE tool are that while it can be used for religious patients, there is no assumption of a faith practice, and is flexible to meet a variety of patient needs. “As far as we are aware, this is the first time the acceptability of the HOPE tool to practitioners has been formally assessed in this way.” Has been added to the discussion.

6. PLOS authors have the option to publish the peer review history of their article ([what does this mean?](https://eur03.safelinks.protection.outlook.com/?url=https%3A%2F%2Fjournals.plos.org%2Fplosone%2Fs%2Feditorial-and-peer-review-process%23loc-peer-review-history&data=05%7C01%7Corla.whitehead%40newcastle.ac.uk%7Cd42241b5cfb64995012308da5933db4f%7C9c5012c9b61644c2a91766814fbe3e87%7C1%7C0%7C637920377623501072%7CUnknown%7CTWFpbGZsb3d8eyJWIjoiMC4wLjAwMDAiLCJQIjoiV2luMzIiLCJBTiI6Ik1haWwiLCJXVCI6Mn0%3D%7C3000%7C%7C%7C&sdata=lkTPKJx2yW0MX5TXrnYo21A6OM0U5UeP%2Bs5f%2FGo8IWc%3D&reserved=0)). If published, this will include your full peer review and any attached files.


If you choose “no”, your identity will remain anonymous but your review may still be made public.


**Do you want your identity to be public for this peer review?** For information about this choice, including consent withdrawal, please see our [Privacy Policy](https://eur03.safelinks.protection.outlook.com/?url=https%3A%2F%2Fwww.plos.org%2Fprivacy-policy&data=05%7C01%7Corla.whitehead%40newcastle.ac.uk%7Cd42241b5cfb64995012308da5933db4f%7C9c5012c9b61644c2a91766814fbe3e87%7C1%7C0%7C637920377623501072%7CUnknown%7CTWFpbGZsb3d8eyJWIjoiMC4wLjAwMDAiLCJQIjoiV2luMzIiLCJBTiI6Ik1haWwiLCJXVCI6Mn0%3D%7C3000%7C%7C%7C&sdata=GgFdpoCuJ76ITLsszzSNvTqGKi8Yb31IOeKqE2it7cc%3D&reserved=0).

Reviewer #1: No
